# Prevalence and socio-demographic determinants of diarrhea among children below 5 years in Bondhere district Somalia

**DOI:** 10.11604/pamj.2021.38.391.21636

**Published:** 2021-04-21

**Authors:** Mahad Dahir Turyare, Japheth Nzioki Mativo, Mary Kerich, Alex Karuiru Ndiritu

**Affiliations:** 1 Department of Nutrition, UNICEF, Mogadishu, Somalia,; 2 Department of Public Health, Jomo Kenyatta University of Agriculture and Technology, Juja, Kenya,; 3 Department of Environmental Health, University of Kabianga, Kericho, Kenya

**Keywords:** Children, diarrhea, prevalence, sociodemographic

## Abstract

**Introduction:**

globally diarrhea is rated as the second leading cause of mortality among children below the age of five years. The highest rates of morbidity and mortality as a result of diarrhea are reported in sub-Saharan Africa and South East Asia. Studies have documented Somalia as among the countries with significant high rates of diarrhea among children below the age of 5 years. The aim of the study was to assess the prevalence and socio-demographic determinants of diarrhea.

**Methods:**

the study employed a descriptive cross-sectional study design where data was collected using semi structured questionnaires. Simple random sampling was employed to identify caregivers that were included in the study. The data collected was analyzed using SPSS version 20 at 95% confidence interval. Both descriptive and regression analysis were carried out. The data was presented using tables and graphs. Ethical clearance was sought from University of Eastern Africa Baraton ethical review committee. Permission and consent were sought from the administrative leadership of Bondhere district and caregivers respectively.

**Results:**

the prevalence of diarrhea among children under 5 years was 22.4%. Socio-demographic factors reported to significantly influence the prevalence of diarrhea among children under years were caregiver education level and number of children under 5 years.

**Conclusion:**

the prevalence of diarrhea among children under 5 years was considerably high. Several socio-demographic factors were associated with diarrhea. The study recommends improvement of education and sensitization of communities on family planning.

## Introduction

Globally diarrhea is the second leading cause of childhood mortality. Children in developing countries are adversely affected by preventable and treatable diseases with modest and financially feasible interventions [1]. Furthermore, in developing countries childhood mortality is almost 10 times higher as compared to the developed nations. There are estimated 1.7 billion cases of diarrhea every year. Consequently, diarrhea accounts for about 525,000 deaths of children under 5 years annually [2]. Sub-Saharan Africa and Southeast Asia accounts for the highest rates of childhood mortality [3]. In Africa it is estimated that children below 5 years´ experience on the minimum five episodes of diarrhea yearly and about 800,000 children succumb to diarrhea annually [4]. In Somalia studies have reported high rates of diarrhea among children below 5 years and is thus reported to be leading cause of childhood morbidity and mortality in this region [5].

Diarrhea is normally characterized by passage of three or more loose or liquid stools daily. Diarrhea is normally an outcome of intestinal tract infections which are normally caused by viral, bacterial or parasitic infestation. Diarrhea is normally spread through drinking or eating contaminated water and food and also from one person to another due to poor hygiene [2]. In most African countries, diarrhea tops the list of waterborne infections as a consequence of poor sanitation. The incidences of diarrhea are more rampant within the first two years of life and declines as the child progresses in age [4]. Studies have documented that diarrhea is not purely medically related but is as well associated with economic, social, environmental and behavioral factors [6]. Some of the socioeconomic factors influencing diarrhea includes; low maternal education levels, poor sanitation, overcrowding and inadequate access to clean and drinkable water [7]. Similarly based on a study conducted in Ethiopia, father’s occupation, educational level and age of the child were cited as key factors influencing diarrhea among children below 5 years [6]. Studies have also documented the use of infants feeding bottles, poor hand washing, poor disposal of faecal matter and failure to breastfeed children up to the age of one year as factors influencing diarrhea among children below 5 years [8,9]. This study therefore aims at investigating the prevalence and socio-demographic factors influencing diarrhea among children below the age of 5 years in Bondhere district, Somalia which are not extensively documented to the best of our knowledge.

## Methods

**Study site:** the study was conducted in Bondhere district. Bondhere district is located in the southeastern part of Banaadir region Somalia. Bondhere district is an administrative region and its coordinates are 2°1'59.999"N, 45°21'0.000"E. Bondhere district is one of the districts in the Banaadir region. The region has a population size of 1,650,227 with an inclusion of 369,288 internally displaced persons (IDPs) [10]. The region is bordered by lower Shebelle, middle Shebelle and the Somalia sea.

**Study design:** the research employed descriptive cross-sectional design which helped in determining factors influencing adoption of hygienic practices associated with diarrhea among children under 5 years in Bondhere district.

**Experimental procedure:** data on socio-demographic factors and rate of diarrhea was collected using a semi structured questionnaire during the period between May and July 2019 from caregivers of children below 5 years. The questionnaires were interviewer administered by data clerks who had prior experience and training on data collection. The study was ethically approved by University of Eastern Africa Baraton. Permission and consent was sought from the administrative leadership of Bondhere district and caregivers respectively. A sample size of 246 caregivers was computed using fisher´s formula and clustered simple random sampling was used to enroll participants in the study. Bondhere district was clustered into the 4 sub districts i.e. Nasib Budo, Yusuf Al-Konwnayn, Daljirka and Sinay. Study participants were then simple randomly selected from the four sub districts.

**Data analysis and presentation:** data was cleaned, coded and data entry done. The data was analyzed using Statistical Package for Social Scientists (SPSS version 20.0). Binomial regressions were computed to show the interactions between the socio-demographic variables and prevalence of diarrhea. The regression analysis was done at 95% confidence level.

## Results

**Socio-demographic characteristics:** in this study 35.8% of the caregivers that were interviewed were household heads while 64.25% were not household heads. Seventy-six-point one percent (76.1%) of the caregivers were married, 13.4% were divorced, 6.0% were single and 4.5% were widowed. Sixteen-point four percent (16.4%), 6.2% and 22.4% had one, two to three and three and above children under 5 years respectively. Of the children below 5 years, 53.5% were male and 46.5% were female. Forty-point three percent of the caregivers had no formal education, 25.4% of the caregivers had attained primary education, and 24.5% and 9.0% of the caregivers had studied up to secondary and tertiary level respectively. Sixty-four-point two percent percent (64.2%) of the caregivers were livestock keepers, 8.1% of the caregivers were salaried employees, 8.9% were formal business owners, 15.9% were informal business owners and 2.9% the caregivers were crop farmers (Table 1). In the current study 22.4% of the caregivers reported that their children had experienced diarrhea in the last two weeks before the survey was conducted while the 77.6% of the caregivers reported that their children had not experienced diarrhea in the last two weeks before the survey was carried out.

**Table 1:** socio-demographic characteristics of the caregivers

Variables	Frequency	Percentage
Are you the household head		
Yes	88	35.8
No	158	64.2
**Who is the household head**		
Husband	165	67.2
Mother in law	15	6
Father in law	11	4.5
**Marital status**		
Single	15	6.0
Divorced	33	13.4
Married	187	76.1
Widowed	11	4.5
**Number of children below 5 years**		
One	40	16.4
Two to three	151	61.2
Three and above	55	22.4
**Gender of children below 5 years**		
Male	132	53.5
Female	114	46.5
**Highest level of education**		
No formal education	99	40.3
Primary	63	25.4
Secondary	62	25.4
Tertiary	22	9.0
**Primary occupation**		
Livestock keeping	158	64.2
Salaried employee	20	8.1
Formal business owner	22	8.9
Informal business owner	39	15.9
Crop farming	7	2.9

Binary regression was computed to determine socio-demographic factors influencing prevalence of diarrhea among children under 5 years. The Nagelkerke R Square value of the model was 0.566 which implies that there was a combined variation of 56.6% of the factors influencing prevalence of diarrhea (Table 2). The model was significant with a p value of 0.010 as shown in Table 3 thus the relationship between the socio-demographic variables and prevalence of diarrhea was significant. The regression analysis shows that socio-demographic factors that had significant influence on prevalence of diarrhea among children below 5 years were number of children below 5 years (0.049) and level of education (p = 0.037) while household head (p = 0.998), marital status (p = 0.92), occupation (p = 0.24) and monthly income (p = 0.068) had no significant influence on prevalence of diarrhea (Table 4).

**Table 2:** regression analysis model summary

Step	-2 Log likelihood	Cox & Snell R Square	Nagelkerke R Square
1	18.968^a^	0.303	0.566

a. Estimation terminated at iteration number 6 because parameter estimates changed by less than 0.001.-

**Table 3:** regression analysis Omnibus tests of model coefficients

		Chi-square	df	Sig.
Step 1	Step	16.932	6	0.010
	Block	16.932	6	0.010
	Model	16.932	6	0.010

**Table 4:** regression analysis of socio-demographic factors influencing diarrhea

		B	S.E.	Wald	df	Sig.	Exp(B)	95% CI for EXP(B)	
								Lower	Upper
Step 1^a^	Household head	-6.303	3137.653	0.000	1	0.998	0.002	0.000	1.4351
	Marital status	0.819	8.110	0.010	1	0.920	2.267	0.000	8.910
	Number of children <5 yrs	-3.219	1.770	3.309	1	0.049	1.040	1.001	2.284
	Level of education	1.503	0.719	4.366	1	0.037	4.494	1.098	18.401
	Occupation	0.709	0.603	1.383	1	0.240	2.032	0.623	6.625
	Monthly income	0.668	0.366	3.326	1	0.068	1.950	0.951	3.998
	Constant	-2.263	3137.738	0.000	1	0.999	0.104		

Variable(s) entered on step 1: household head, marital status, children < 5yrs, level of education, occupation, monthly income

## Discussion

The dominant diseases affecting children below the age of 5 years in developing countries includes: diarrhea, malnutrition and respiratory infections [11]. Somalia is catastrophically affected by diarrhea with significant number of children being affected. The incidences of diarrhea in Somalia tends to increase with intensification of drought conditions [12]. The overall two weeks prevalence of diarrhea among children under 5 years in this study was 22.4%. Similarly based on a study conducted in Eastern Ethiopia, the prevalence of diarrhea among children below the age of 5 years was 22.5% [8]. Equally in a study done in Northwest Ethiopia the prevalence of diarrhea was reported as 21.5% [1]. However, the prevalence of diarrhea reported in this study was considerably higher than 19.5% reported in Somalia based on a 2011 Ethiopian Demographic and Health Survey [13]. Relatedly based on a study conducted in Burundi higher prevalence (32%) of diarrhea were reported as compared to the values reported by this study [3]. The variation in prevalence of diarrhea could be potentially attributed to differences in study area, infant and young children feeding and hygiene and sanitation [11].

Socio-demographic factors reported to significantly influence the prevalence of diarrhea among children below the age of 5 years were: number of children below 5 years and level of education. Based on this study over 80% of the caregivers had over two children below the age of 5 years thus the considerable demand for maternal care could result to lower adherence on hygiene and sanitation by the caregivers hence resulting to diarrhea. Similarly based on a study conducted in Northwest, Ethiopia having more than two children under the age of five years was cited as a determinant of diarrhea [14]. Relatedly based on studies conducted in Egypt and Eritrea the prevalence of diarrhea was reported to increase with the increase in number of children below the age of years [15,16]. According to Kawakatsu *et al*. [17] high prevalence of diarrhea was reported among children of illiterate caregivers. In this study over two thirds of the caregivers were reported to have primary as their highest level of education. Relatedly studies have cited that, children of mothers with an education below secondary level are more likely to experience diarrhea [18]. Studies have documented that maternal education is crucial to population health and its impacts are evident at individual and community level [19]. Additionally, studies have cited that well educated caregivers are more likely to have adequate personal hygiene and sanitation and better health seeking behavior which cumulatively potentially reduces the prevalence of diarrhea [20].

The current study documents that marital status, occupation and monthly income had no significant influence on the prevalence of diarrhea. Similarly based on a study conducted in Ethiopia, occupation and monthly income had no significant association with diarrhea among children under 5 years [21]. However, studies have reported significant association between family income and the occurrence of diarrhea among children below 5 years [22]. For instance, studies have reported that caregivers with high income will tend to seek modern treatment for diarrhea for their children [23]. Relatedly based on a study done in sub-Saharan Africa occupation was reported to significantly influence prevalence of diarrhea [24]. The disparities between the current study findings and other studies could be attributed to differences in maternal attention and study designs [21].

## Conclusion

The prevalence of diarrhea among children under the age of the last two weeks prior to the survey was 22.4%. Socio-demographic factors that were reported to significantly influence the prevalence of diarrhea were; number of children below the age of five years and caregiver education level. Therefore, the Somalia government and other partners in the health sector should devise strategies to educate people on family planning and ensure accessibility of formal and informal education.

### What is known about this topic

- Strategies being implemented by government and other partners to reduce diarrhea.

### What this study adds

- Prevalence of diarrhea among children under 5 years in Bondhere district;
- Caregivers’ socio-demographic determinants of diarrhea;
- The way forward in reducing the prevalence of diarrhea among children under 5 years.
